# The Foe You Know: Observations of Interspecific Interactions Between Small Cetaceans and Northern Resident Killer Whales ( *Orcinus orca* ) in the Northeast Pacific

**DOI:** 10.1002/ece3.71444

**Published:** 2025-06-02

**Authors:** Brittany C. Visona‐Kelly, Lance G. Barrett‐Lennard

**Affiliations:** ^1^ Ocean Wise Conservation Association Whales Initiative Vancouver British Columbia Canada; ^2^ Raincoast Conservation Foundation Cetacean Conservation Research Program Sidney British Columbia Canada

**Keywords:** behavior, dolphin, drones, killer whale, mixed‐species groups, porpoise

## Abstract

We documented the first aerial observations of interspecific interactions of fish‐eating, northern resident killer whales ( *Orcinus orca* ) with two small cetacean species, Dall's porpoises ( *Phocoenoides dalli* ) and Pacific white‐sided dolphins ( *Lagenorhynchus obliquidens* ), off northeastern Vancouver Island, BC, Canada. Specifically, we used drone‐collected data to compare observations of porpoises and dolphins interacting with northern resident killer whales to understand factors which may promote interspecific interactions. From 2018 to 2021, 42 interactions were opportunistically recorded of Dall's porpoises (19%), Pacific white‐sided dolphins (74%), and both species (7%) approaching groups of traveling, milling, socializing, and resting northern resident killer whales. The mean group size of killer whales during interactions with dolphins (an average of 8 killer whales) and porpoises (an average of 3 killer whales) was significantly different. Porpoises interacted only with small groups (*n* ≤ 5) of killer whales, while dolphins interacted with larger groups (*n* ≥ 25). This suggests that the likelihood of interaction by each species is influenced by killer whale group size. However, the age and sex composition of killer whale groups, as well as the number of small cetaceans present, were found to have no significant effect on interaction patterns. Additionally, we never observed killer whales initiating interactions. We discuss several adaptive benefits, including antipredator, foraging, hydrodynamic, and social advantages, to dolphins and porpoises that may drive the observed interactions. The benefits of interactions appeared to be predominantly antipredator‐focused and species‐specific, with both species possibly also benefiting from increased hydrodynamic efficiency while swimming. While opportunistic, our observations provide detailed accounts of non‐predatory interactions between small cetacean species and northern resident killer whales. Future studies focusing on the drivers of mixed‐species group formation in the North Pacific are needed, and we suggest that these utilize drones as a platform for data collection.

## Graphical abstract

This study presents the first aerial observations and the most detailed accounts to date of interspecific interactions between northern resident killer whales and two small cetacean species, Dall's porpoises and Pacific white‐sided dolphins, off northeastern Vancouver Island, BC, Canada from 2018 to 2021. The findings suggest that these interactions may offer adaptive benefits to small cetaceans, including protection from Bigg's killer whales, opportunities to steal fish or scavenge scraps, and enhanced hydrodynamic efficiency.

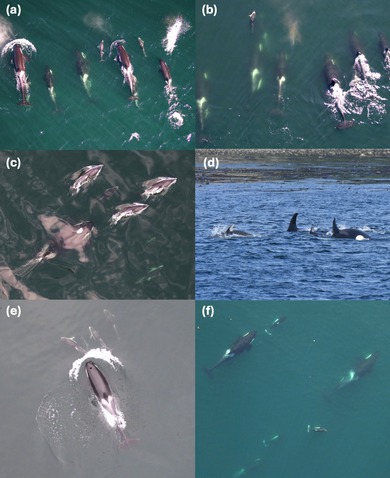

## 1 Introduction

Interactions between two or more species, known as “interspecific interactions,” that are not associated with predation, are common in the animal kingdom and have been observed in species of fish (Paijmans et al. 2019, 2020), amphibians (Ferrari and Chivers 2008), reptiles (Escobedo‐Galvan et al. 2019; Torres et al. 2024), birds (Thiollay 1999; Sridhar et al. 2009; Mangini et al. 2023), and mammals (Peres 1993; Latham 1999; Chapman and Chapman 2000). Studies often focus on the benefits or detriments of these interactions on participating species (e.g., Gautier‐Hion et al. 1983; Sridhar et al. 2009; Paijmans et al. 2019) and utilize long‐term or continuous observational datasets, collected through either laboratory experiments (i.e., for fish and amphibian species; e.g., Pitcher et al. 1982; Godin and Davis 1995; Ferrari and Chivers 2008), or field surveys (e.g., Chapman and Chapman 2000; Paijmans et al. 2020).

One or all species involved in interspecific interactions may directly benefit from interacting or forming a mixed‐species group. For example, three subspecies of *Cercopithecus* monkeys all benefit from interactions by gaining access to species‐specific foraging areas and antipredation tactics (Gautier‐Hion et al. 1983), whereas the communal egg‐laying of the ornate slider turtle ( *Trachemys ornata* ) in American crocodile ( *Crocodylus acutus* ) nests suggests one‐sided benefits (Escobedo‐Galvan et al. 2019). While both diverse and complex, the primary drivers of interspecific interactions and mixed‐species group formation are typically categorized as antipredator, foraging, or social advantages (Stensland et al. 2003; Syme et al. 2021). Specific advantages that may motivate individuals to interact include access to increased foraging opportunities (Clua and Grosvalet 2001; Jourdain and Vongraven 2017), a reduction in predation pressure or improved predator avoidance (Peres 1993; Lehtonen and Jaatinen 2016; van Langevelde et al. 2022), or enhanced fitness (Herzing and Johnson 1997; Hill et al. 2017; Mrusczok et al. 2023). Aquatic species may also benefit from a reduction in the energetic costs of locomotion through hydrodynamic hitchhiking (Xu et al. 2021).

Interspecific interactions in cetacean species are commonly observed worldwide, particularly within the Odontocete family *Delphinidae* (Herzing and Johnson 1997; Psarakos et al. 2003; Bearzi 2005; Crossman et al. 2016; Eierman et al. 2019; Syme et al. 2021, 2023; Mrusczok et al. 2023). Behavioral interactions with killer whales ( *Orcinus orca* ), an apex predator in many marine ecosystems, have been recorded for more than 20 cetacean species (Jefferson et al. 1991; Visser 1999). While a majority of interspecific interactions observed between two delphinid species are generally mutualistic or commensal (e.g., Herzing and Johnson 1997; Syme et al. 2023), most documented killer whale interspecific interactions with other species are predatory, with only a few reports of non‐predatory interactions, particularly harassment (Jefferson et al. 1991; Ford et al. 1998) and play (Hawke 1989).

In some regions of the world, two or more eco‐typically distinct killer whale populations coexist. One such example is the northeast Pacific, where three ecotypes of killer whales overlap in range: fish‐eating residents (northern and southern populations), mammal‐eating Bigg's (formerly referred to as *transients*), and fish‐ and shark‐eating offshores (Ford et al. 1998, 2011). These ecotypes do not associate or interbreed (Barrett‐Lennard 2000), have different vocal behaviors and repertoires of stereotyped calls (Ford 1989), and pose different levels of risk to other cetaceans (Ford et al. 1998).

Dall's porpoises ( *Phocoenoides dalli* ; hereinafter referred to as “porpoises”) and Pacific white‐sided dolphins ( *Lagenorhynchus obliquidens* ; hereinafter referred to as “dolphins”) are two cetacean species commonly preyed on by Bigg's killer whales. Although resident killer whales visually resemble Bigg's, both of these small cetacean species are known to approach and interact with residents (Jefferson et al. 1991; Ford et al. 1998; A. Morton 2000). While these interactions are typically non‐predatory, there have been rare accounts of resident killer whales harming (e.g., striking or ramming) and even fatally wounding porpoises (Jefferson et al. 1991; Ford et al. 1998; Giles et al. 2023), indicating that associating with residents is not without risk.

Despite North Pacific killer whales being extensively studied (Forney et al. 2006; Morin et al. 2024; Williams et al. 2024), reports of non‐predatory interactions between killer whales, dolphins, and porpoises have been sparsely documented in the literature (Jefferson et al. 1991; Ford et al. 1998; Giles et al. 2023), likely due to the opportunistic nature of interactions. Only recently have interactions between porpoises and killer whales from the southern resident population been summarized in detail using sightings compiled from the last 45 years (Giles et al. 2023). Potential drivers of these interactions have been proposed (Giles et al. 2023) but are thought to be culturally specific to the southern residents and remain poorly understood outside the population.

The recent adoption of drones in behavioral studies (e.g., Torres et al. 2018; Álvarez‐González et al. 2023) opens the door to viewing interspecific interactions between cetacean species from a new perspective. Previously, observations of cetacean interspecific interactions were limited to land or vessel‐based platforms, resulting in a lack of sub‐surface behavioral observations. Here, we provide details of porpoise and dolphin interactions with northern resident killer whales off northeastern Vancouver Island, (British Columbia, Canada) documented opportunistically over four years while conducting drone‐based health assessments of killer whales. The range of northern resident killer whales spans the entirety of the British Columbian coast, from Alaska, USA, in the north to Washington State, USA, in the south, with the Johnstone Strait area identified as critical habitat for the population (Ford et al. 2000, 2017; Fisheries and Oceans Canada 2018). In addition to being a hotspot for northern resident killer whales, this area is often used by Dall's porpoises and Pacific white‐sided dolphins. Previous reports of interactions between these species have been limited and have never been observed or summarized from aerial observations. The aim of this study was to describe interspecific interactions between northern resident killer whales and porpoises and/or dolphins, and to explore potential species‐specific drivers that may promote such interactions.

## 2 Materials and Methods

### 2.1 Study Area and Data Collection

**FIGURE 1:**
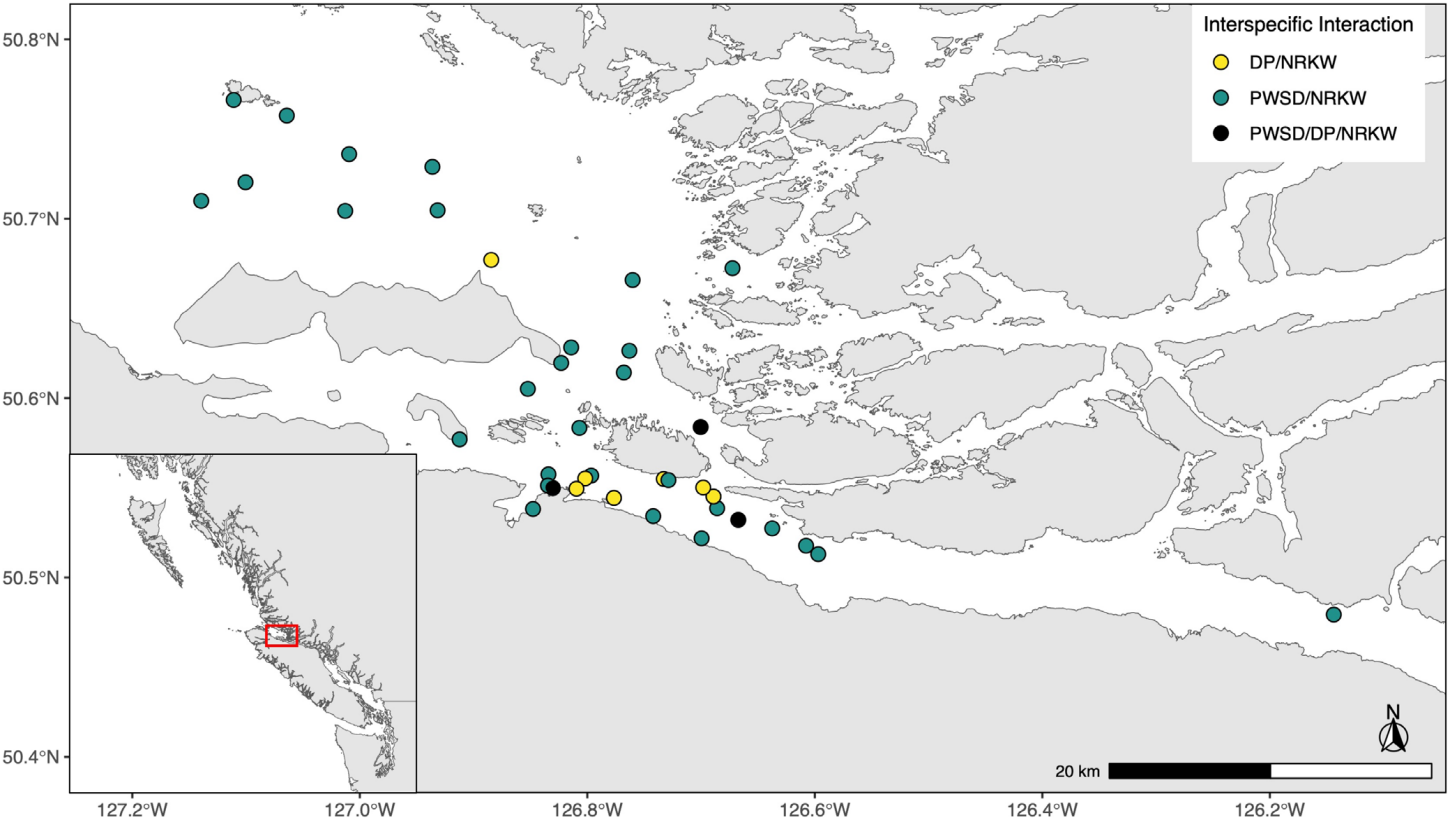
Observations of interspecific interactions of Dall's porpoises (DP, yellow), Pacific white‐sided dolphins (PWSD, turquoise), and both species (black) with northern resident killer whales (NRKW) recorded during annual drone‐based field surveys between July and September from 2018 to 2021 in Johnstone Strait, BC, Canada.

Between July and early September of 2018 to 2021, drone‐based field work was conducted in the vicinity of Johnstone Strait (British Columbia, Canada; Figure 1). The primary purpose for data collection was to collect high‐resolution aerial images for a photogrammetric assessment of killer whale body condition and inferred health. However, during this work, interactions between killer whales and dolphins and/or porpoises were frequently observed, and these were then documented through aerial imagery and field notes to allow studies of interspecific interactions.

Aerial images and videos were collected using a DJI Matrice 200 Unmanned Aerial Vehicle (UAV, drone) equipped with a Zenmuse X5S camera and an Olympus M. Zuiko 25 mm *f*/1.8 lens. The drone was launched and retrieved from an 8.2‐m‐long research vessel positioned 200 m or more away from the whales and flown on short‐duration flights (approximately 20 to 25 min) at altitudes of 25 to 30 m. Due to power limitations with the drone, a maximum of 12 flights (mean = 4.5 flights/day, range 1 to 12) were conducted per day (5–12 h) and were carried out consecutively when weather allowed (i.e., sea state ≥ Beaufort 4 and wind speed ≥ 15 knots). Flight duration was limited to a total of 30 min per individual per day as specified in our research licenses. The taking of still images for photogrammetry was prioritized, but video was recorded when killer whales and other species were seen interacting. All imagery was accompanied by field notes that included encounter details such as date, time, species presence, number of individuals, and species behaviors.

### 2.2 Photo Identification

Lateral photo‐identification images of the killer whale's dorsal fins were collected during all flights using a Nikon D810 digital SLR camera equipped with an 80–400 mm telephoto lens to aid in identifying individuals present. Individual killer whales of known age and sex were identified by matching saddle patch scars and pigmentation patterns to published photo‐identification catalogs (Towers et al. 2020) following standard photo‐identification protocols for killer whales in the North Pacific (Bigg et al. 1976, 1986; Ellis et al. 2011; Towers et al. 2019, 2020).

### 2.3 Data Analysis

#### 2.3.1 Identifying Interspecific Interactions

A combination of imagery and field notes were used to describe species behaviors and interaction details; QuickTime Player (1154.4.2) was used to visually inspect 4 K videos while images were scanned in succession using ACDSee Photo Studio (7.0.1840). For the purpose of this study, an “interspecific interaction” (hereinafter referred to as an “interaction”) was defined as any encounter where a dolphin or a porpoise appeared to be within 200 m of one or more killer whales and was attracted by killer whale presence, rather than by localized concentrations of prey (following Jefferson et al. 1991 and Stensland et al. 2003). Dolphins and porpoises are generalist predators, preying on a variety of species, primarily high‐energy schooling fish (Heise 1997; Morton 2000; Nichol et al. 2013), whereas resident killer whales are specialists, with Chinook salmon comprising over 70% of their diet (Ford et al. 2010). Given the difference in prey species and the absence of large aggregations of birds and pinnipeds that utilize high‐density prey patches (Au and Pitman 1986; Clua and Grosvalet 2001; Bearzi 2006) during encounters, it was assumed that interactions occurred independently of prey. Interactions were considered to have ended when no species remained within 200 m of each other, as we observed no evidence of interactions or coordinated behavior at this distance, and dolphins and porpoises would typically leave the area once this distance was reached.

When possible, as interactions were documented opportunistically, we recorded: (1) duration of interaction; (2) number of individuals present for each species; (3) identity of individual killer whale(s); (4) behavioral state of each species before and during an interaction (see definitions in Appendix A); and (5) potential additional information (e.g., manner in which members of one species approached the other, formation and position of individuals and species, presence of prey remains, etc.). All statistical analyses were conducted in RStudio (1.2.5042; RStudio Team 2020).

#### 2.3.2 Investigating Species‐Specific Characteristics of Interactions

To identify potential species‐specific characteristics of porpoise and dolphin interactions with killer whales, the following parameters were quantified for each identified interaction: (1) duration—length of interaction in minutes; (2) total killer whales present—maximum number and potential identification of individual killer whales; (3) total number of small cetaceans—mean number of dolphins and porpoises present based on the range of group size estimates; and (4) killer whale group composition—age and sex of killer whales present based on demographic information in the photo‐identification catalogs.

After individuals were photo‐identified, killer whale groups were classified by age‐sex composition for each interaction. Age classification was evaluated at three levels: (1) juveniles—individuals ages 0 to 12, as the average age of maturity is 13 for male killer whales and females typically have their first calf at 14 (Olesiuk et al. 2005), (2) adults—individuals aged 13 and older, or females younger than 13 years of age that were pregnant or had their first calf, and (3) mixed—combination of juveniles and adults. Group composition during interactions was also evaluated as: (1) female—all individuals female, (2) male—all individuals male, and (3) mixed—combination of male and female individuals or animals of unknown sex present. The frequency of the age‐sex composition of killer whale groups was assessed for all porpoise and dolphin interactions with killer whales.

Regional preference differs between the three northern resident clans (pods of resident killer whales with shared call elements, referred to as A, G, and R clan; Ford 1991), with A clan having the greatest affinity for Johnstone Strait (Ford et al. 2000). Specifically, the matrilines (group of associating individuals with a common mother or grandmother) of A1, A4, A5, and C1 pods (large group consisting of multiple related matrilines) often travel together and frequently visit the study area during summer months (Ford et al. 2000). Since interactions were collected opportunistically, with a bias towards more commonly sighted groups, no comparisons were made between killer whale matrilines, pods, or clans observed in interactions.

A Shapiro–Wilk normality test was used to assess the distribution of the data (Shapiro and Wilk 1965). Given the non‐parametric nature of the dataset, a Wilcoxon rank‐sum test (Rey and Neuhäuser 2011) was applied to compare the duration and number of individuals present during porpoise‐ and dolphin‐killer whale interactions. Any interactions in which all three species were present were excluded. Significance was considered at *α* = 0.05.

#### 2.3.3 Frequency and Change of Behavioral States

For each interaction where behavioral data was available, all species were assigned a single or combination of behavioral state categories (defined in Appendix A) before and during the interaction. The frequency of behavioral states was calculated separately for northern resident killer whale interactions with porpoises and dolphins. If two or more behavioral states were recorded for a species before or during an interaction (e.g., traveling and milling during an interaction), the frequency was calculated by considering the equal proportion of behavioral states recorded, allowing comparative analysis between interactions. Comparisons of before and during frequencies were considered for interactions where only dolphins or only porpoises were present with killer whales and interactions where behavioral states were recorded. Interactions where both porpoises and dolphins were present were not considered, as it was not possible to determine whether a behavioral change was the result of interacting with killer whales or the other species. If recorded behaviors before and during an interaction differed (e.g., milling to foraging, grouped to dispersed, slow travel to fast travel), a change in behavioral state was recorded.

#### 2.3.4 Approach Direction and Swimming Position

When observed, the species that initiated interactions and the approach direction of the species was recorded and summarized for both porpoise and dolphin interactions with northern resident killer whales. A species was described as approaching another from either the front, side, or behind. If the direction of approach was not recorded, it was categorized as an “unknown” approach direction.

Additionally, the swimming positions of porpoises and dolphins relative to the killer whales were also noted whenever possible. These positions were defined as porpoises/dolphins swimming ahead, alongside, behind, overhead (i.e., above), through (i.e., in‐between two or more whales), and/or underneath (i.e., below) a killer whale or group of killer whales. Porpoises and dolphins could occupy one or more swimming positions during an interaction. To quantify the types of positions observed, a count of one swimming position was assigned for each interaction. If multiple positions were observed, the swimming position for that interaction was determined by calculating the equal proportion of all recorded positions, ensuring the total for each interaction equaled one.

## 3 Results

### 3.1 Interspecific Interactions

Interactions between northern resident killer whales and dolphins and porpoises were observed annually within the study area during 117 field days, nearly evenly distributed from 2018 to 2021 (Figure 1, Table 1). A total of 42 killer whale interactions were observed: 74% (31 out of 42) involved Pacific white‐sided dolphins, 19% (8 out of 42) involved Dall's porpoises, and 7% (3 out of 42) involved both species. Due to the low number of encounters with porpoises, dolphins, and killer whales, and because our project focused on species‐specific characteristics of interactions, the interactions involving all three species were excluded from further data analysis. Interactions included individuals from 28 northern resident matrilines from all three clans (see Appendix B for encounter details). Bigg's killer whales were sighted 36 times during the study period (Appendices C, D, E), but no interactions with resident killer whales were observed.

**TABLE 1:** Interspecific interactions between northern resident killer whales (NRKW) and Dall's porpoises (DP) and Pacific white‐sided dolphins (PWSD) recorded July to September from 2018 to 2021 off northeastern Vancouver Island, BC, Canada.

Year	Number of field days	Number of interactions	Number of NRKW‐DP interactions	Number of NRKW‐PWSD interactions	Number of NRKW‐DP‐PWSD interactions
2018	28	10	0	9	1
2019	29	11	4	7	0
2020	28	11	1	9	1
2021	32	10	3	6	1
Total	117	42 (100%)	8 (19%)^a^	31 (74%)^a^	3 (7%)^a^

^a^ Percentage of species‐specific interactions relative to the total interspecific interactions observed during the study period.

### 3.2 Species‐Specific Characteristics of Interactions

#### 3.2.1 Duration

**FIGURE 2:**
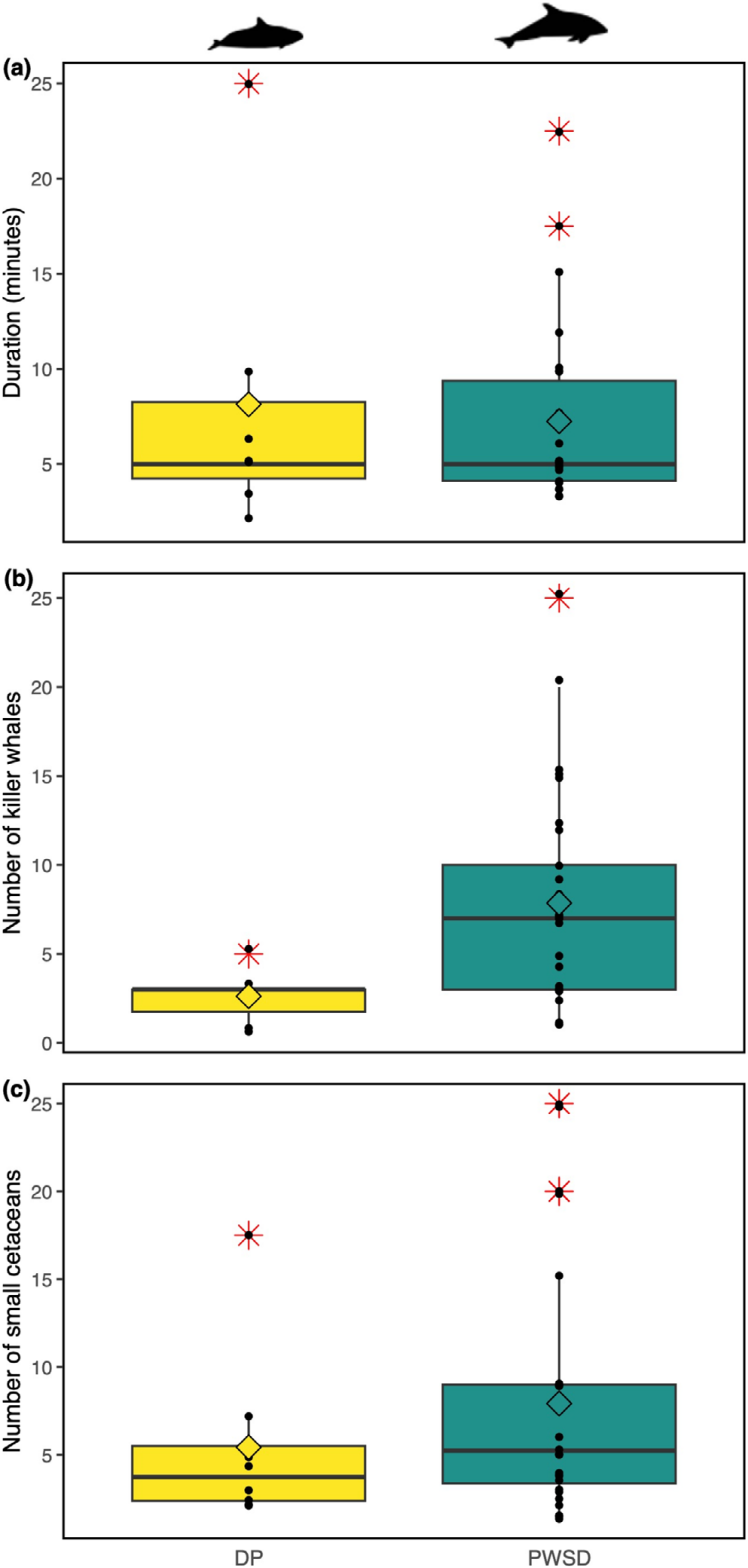
Boxplot (box: Interquartile range, whiskers: 1.5 times the interquartile range, black bar: median, black points: individual interactions, diamond: mean, red star: outliers) of (a) duration (*n*_DP_ = 7, *n*_PWSD_ = 26), (b) number of killer whales (*n*_DP_ = 8, *n*_PWSD_ = 29), and (c) number of small cetaceans (*n*_DP_ = 8, *n*_PWSD_ = 28) recorded during northern resident killer whale interspecific interactions with Dall's porpoises (DP) and Pacific white‐sided dolphins (PWSD) off northeastern Vancouver Island, BC, Canada from 2018 to 2021. Two extreme outliers were removed from boxplots: a 105‐min PWSD‐NRKW interaction, and a PWSD‐NRKW interaction involving an estimated > 250 individuals.

The duration of interactions varied over the study period for both porpoise (mean duration = 8.14 min ±7.85, mode = 5 min, range = 2 to 25 min, *n* = 7; duration not recorded for 1 interaction) and dolphin (mean duration = 10.87 min ±19.40, mode = 5 min, range = 3.5 to 105 min, *n* = 27; duration not recorded for 4 interactions) interactions with northern resident killer whales (Figure 2). The longest interactions recorded were between dolphins and killer whales; one notable interaction lasted over 1.5 h (see Appendix B for details). The shortest duration observed (2 min) was during a porpoise–killer whale interaction, but short interactions (< 5 min) were often observed for both porpoise and dolphin interactions (*n*_porpoise_ = 2 out of 7, *n*_dolphin_ = 8 out of 27). These interactions typically involved the two species traveling by or through the group of killer whales before continuing through the area (see Appendix B for details). No difference in mean duration was found between porpoise and dolphin interactions (Wilcoxon test, *W* = 89.5, *n*_porpoise_ = 7, *n*_dolphin_ = 26*, *p* = 0.96; Figure 2; *105 min interaction removed from comparison; Figure 2).

#### 3.2.2 Group Size

The minimal group size of porpoises and dolphins engaged in interactions with northern resident killer whales was similar for both species, with interactions observed when only 1–2 individuals were present (Table 2). The largest groups of small cetaceans were noted during dolphin interactions, with an estimated > 250 individuals present during one encounter (Table 2, Appendix B). Killer whale group size was largest during dolphin interactions, compared to porpoise interactions (Table 2). A comparison of porpoise and dolphin interactions with killer whales revealed that the mean number of killer whales present differed significantly between species (Wilcoxon test, *W* = 43, *n*_porpoise_ = 8, *n*_dolphin_ = 29, *p* = 0.01; Figure 2). However, no significant difference was found in the mean number of porpoises and dolphins present (Wilcoxon test, *W* = 81, *n*_porpoise_ = 8, *n*_dolphin_ = 28*, *p* = 0.24; Figure 2; *group size not recorded for 2 interactions).

**TABLE 2:** Number of northern resident killer whales (NRKW), Dall's porpoises (DP), and Pacific white‐sided dolphins (PWSD) observed during interspecific interactions recorded from 2018 to 2021 around Vancouver Island, BC, Canada.

Species	Interaction	*n*	Mean	Mode	Minimum	Maximum
NRKW		40^a^	6.9	3	1	25
	DP	8	2.63	3	1	5
	PWSD	29^a^	7.86	7	1	25
DP		8	5.4	2	2	17.5
PWSD		29^a^	7.9^b^	9	1.5	250^c^

^a^ Number of animals present was not recorded for every interaction.

^b^ Maximum value was removed from the mean calculation.

^c^ Estimate of maximum group size.

#### 3.2.3 Age and Sex Composition

**FIGURE 3:**
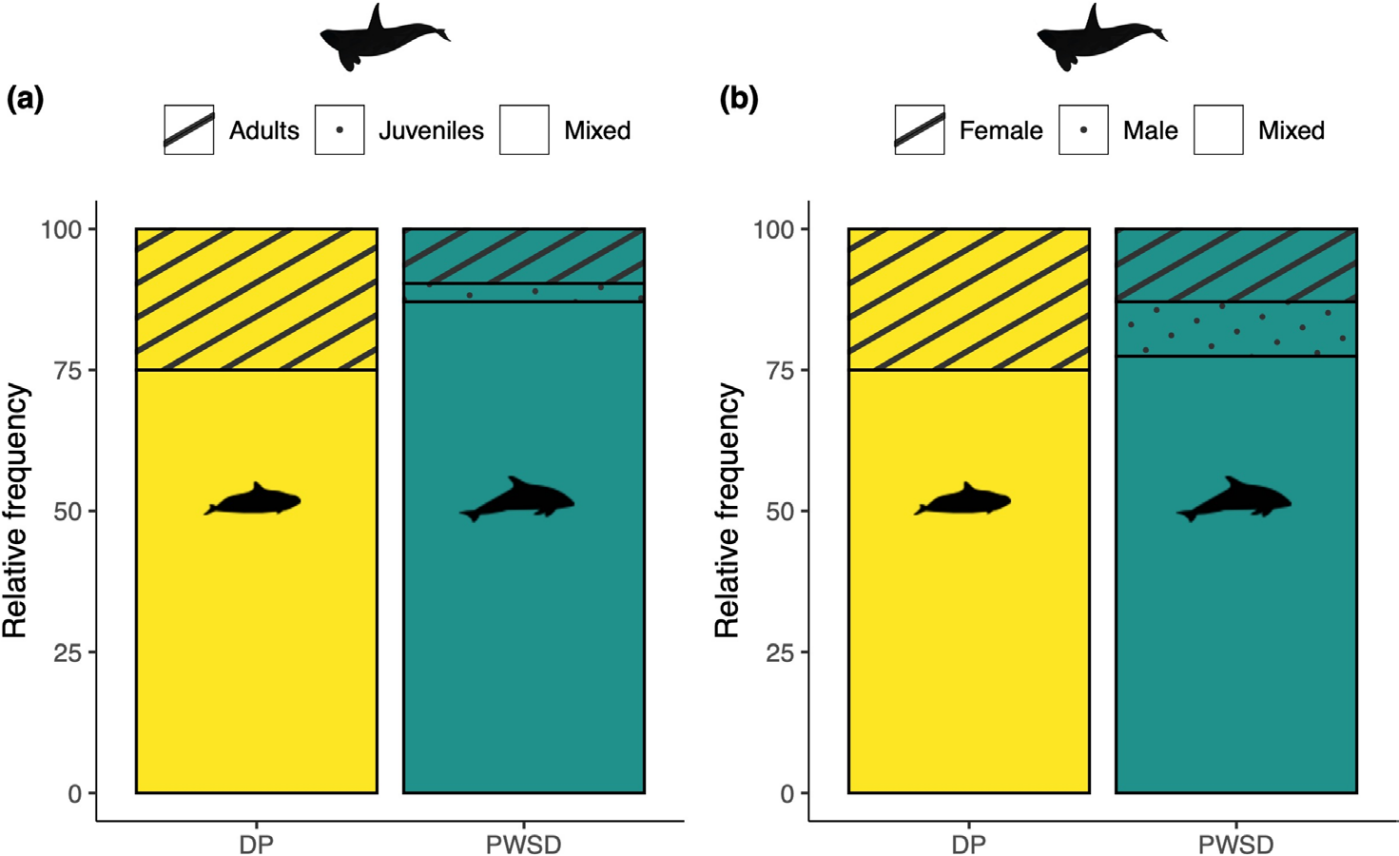
Observed (a) age and (b) sex composition of northern resident killer whale groups involved in Dall's porpoise (DP, *n* = 8) and Pacific white‐sided dolphin (PWSD, *n* = 31) interactions off northeastern Vancouver Island, BC, Canada.

Porpoises and dolphins engaged with northern resident killer whales of all ages and sexes, ranging from calves to mature males and post‐reproductive females (Appendix B). The age and sex composition of killer whale groups present during interactions was similar for porpoise and dolphin interactions, except that juvenile‐ and female‐only groups were only observed with dolphins (Figure 3). Mixed groups of juvenile and adult killer whales were recorded during 75% (6 out of 8) of porpoise and 87% (27 out of 31) of dolphin interactions. Adult‐only groups were observed in 25% (2 out of 8) of porpoise and 10% (3 out of 31) of dolphin interactions, while juvenile‐only groups were observed in 0% (0 out of 8) and 3% (1 out of 31) of porpoise and dolphin interactions, respectively. Female and male mixed groups comprised 75% (6 out of 8) of porpoise and 77% (24 out of 31) of dolphin interactions. Male‐only groups were not observed during porpoise interactions, but female‐only groups were present 25% (2 out of 8) of the time. For dolphin interactions, male‐only groups were recorded in 10% (3 out of 31) of interactions and female‐only groups in 13% (4 out of 31).

### 3.3 Frequency and Change of Behavioral States

**FIGURE 4:**
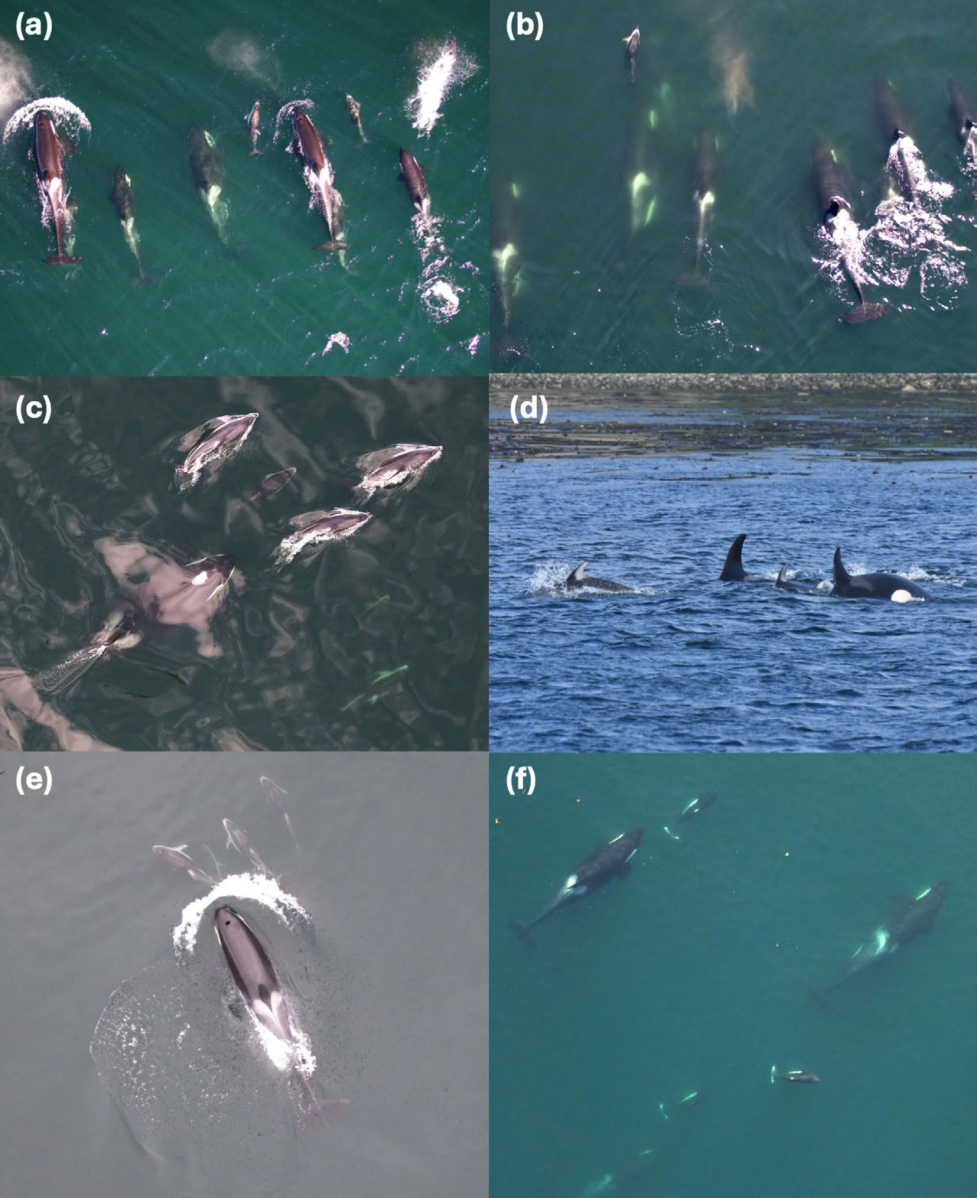
Examples of Pacific white‐sided dolphin (a–e) and Dall's porpoise (f) behavior during interactions with (a) logging, (b) resting, (c–e) milling, and (f) traveling northern resident killer whales in the North Pacific.

**FIGURE 5:**
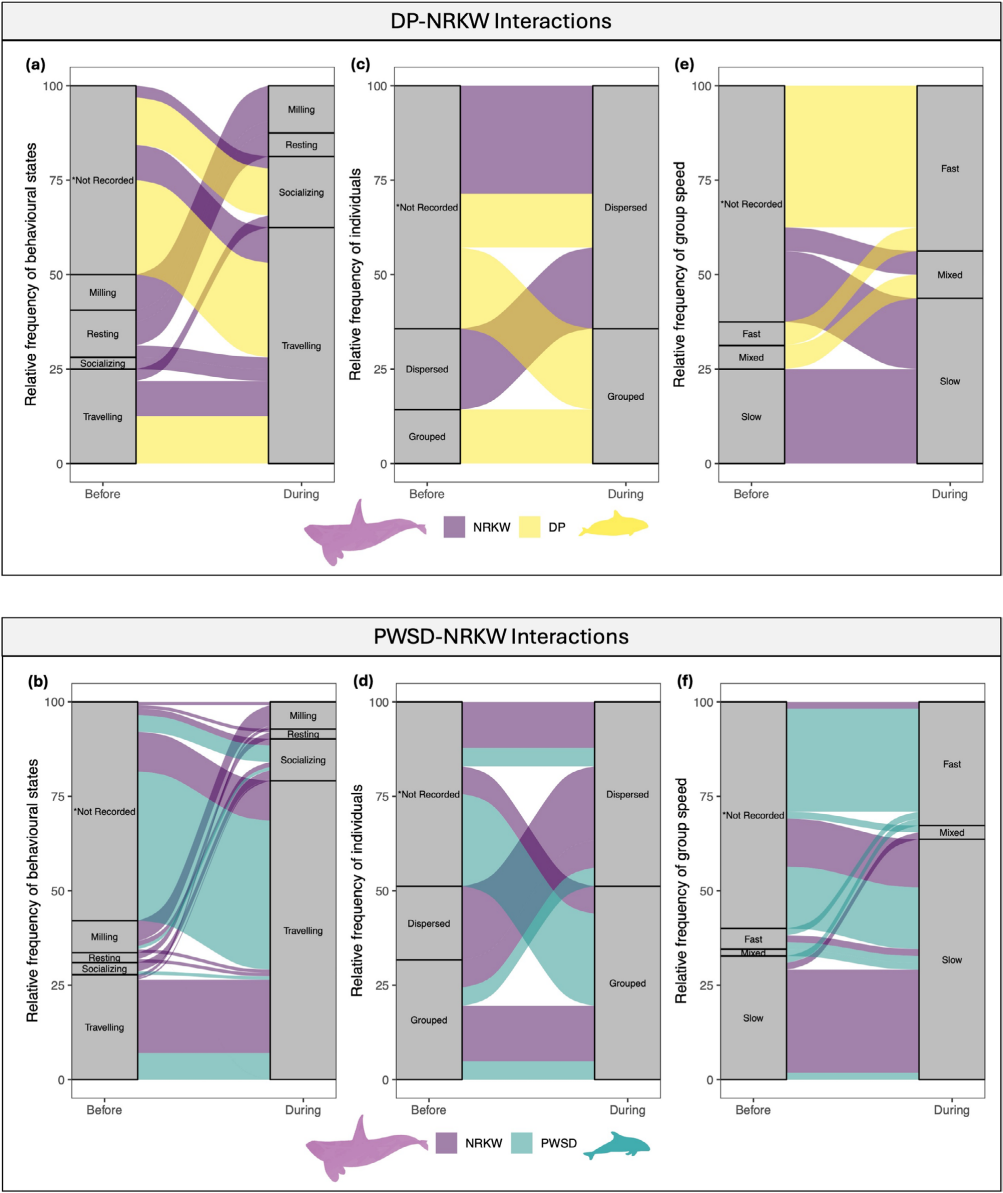
Occurrence of (a, b) behaviors (*n*_NRKW‐DP_ = 8, *n*_DP_ = 8, *n*_NRKW‐PWSD_ = 27, *n*_PWSD_ = 30), (c, d) spread of individuals (*n*_NRKW‐DP_ = 7, *n*_DP_ = 7, *n*_NRKW‐PWSD_ = 25, *n*_PWSD_ = 16), and (e, f) group speed (*n*_NRKW‐DP_ = 8, *n*_DP_ = 8, *n*_NRKW‐PWSD_ = 25, *n*_PWSD_ = 30) of Dall's porpoises (DP, yellow), Pacific white‐sided dolphins (PWSD, turquoise), and northern resident killer whale (NRKW, purple) before and during interactions recorded during 2018 to 2021 off northeastern Vancouver Island, BC, Canada. Characteristics of species before interactions was not always recorded as interspecific interaction data was opportunistically collected.

Porpoises and dolphins engaged in interactions with killer whales in any behavioral state (Appendix B; see Figure 4 and Videos S1–S5 for examples). Killer whales were observed traveling in most porpoise (4 out of 8) and dolphin (18 out of 27) interactions in which behavioral state was recorded (Figure 5). Similarly, porpoises and dolphins were observed traveling during a majority (6 out of 8 and 27 out of 30, respectively) of interactions. When behavior was recorded, traveling was also the most common behavioral state before an interaction occurred for all species (Figure 5). During both porpoise and dolphin interactions, killer whales were typically dispersed, with individuals spread out and moving slowly (Figure 5, Appendix B). In contrast, during interactions with killer whales, porpoises and dolphins were often grouped together, with individuals moving quickly. Group formation and speed for all species were often not recorded before interactions to provide trends of species‐specific behavioral states (Figure 5).

**FIGURE 6:**
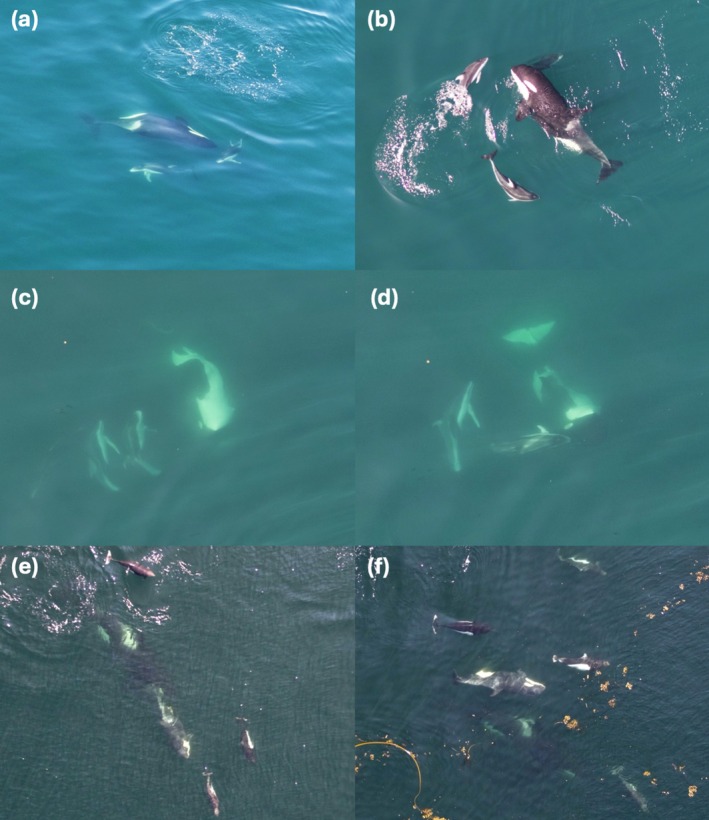
Images of social behavior displayed on September 1, 2018 (a–d) by northern resident killer whale A118 (newborn calf) and three Pacific white‐sided dolphins and on July 8, 2021 (e–h) by northern resident A126 (newborn calf) and 15 to 20 Dall's porpoises off northeastern Vancouver Island, BC, Canada.

**FIGURE 7:**
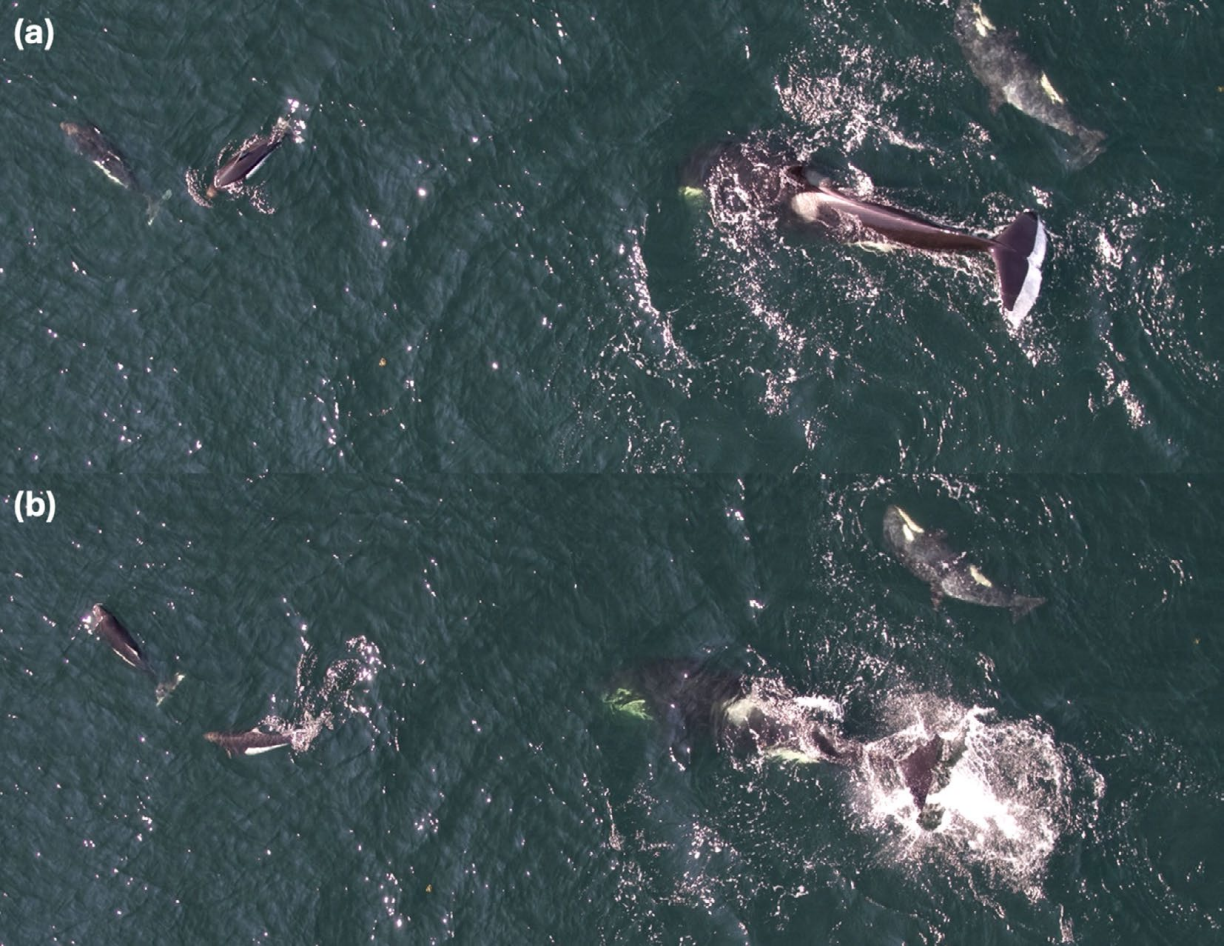
Tail slapping behavior (a, b) exhibited by adult female northern resident killer whale A69 during a Dall's porpoise interaction with her and her newborn A126 on July 8, 2021, in Johnstone Strait, BC, Canada.

Dolphins and porpoises displayed what appeared to be social behavior with young killer whale calves during three encounters (see Figure 6, Video S2, and Appendix B). Two observations of apparent aggressive behavior displayed by killer whales during interactions with porpoises and dolphins were also recorded (see Figure 7, Video S3, and Appendix B). No other observations of visible avoidance by porpoises or dolphins were observed during the study period.

Due to the opportunistic nature of the collected data, the behavioral state before and during interactions was recorded for killer whales in 26 out of 39 interactions, and in 8 out of 39 interactions for dolphins and porpoises. All species displayed a variety of behaviors before and during interactions (Figure 5). Killer whale behavior changed in 42% (11 out of 26) of interactions, while dolphin behavior changed in 60% (3 out of 5) and no change (0 out of 3) was recorded for porpoises (Table 3, see details in Appendix B). Changes typically occurred in the behavior of killer whales during interactions with either species; however, killer whales were also observed changing speed and group formation during dolphin interactions (Figure 5, Table 3). Dolphins changed behavior, speed, and formation during interactions on several occasions where behavioral states were recorded (Figure 5, Table 3).

**TABLE 3:** Summary of difference in behavioral states before and during northern resident killer whale (NRKW), Dall's porpoise (DP), and Pacific white‐sided dolphin (PWSD) interspecific interactions recorded July to September of 2018 to 2021 off northeastern Vancouver Island, BC, Canada. Values include only interactions where both before and during behavioral states were recorded.

Species	Interaction	*n*	No change % (*n*)	Change occurred % (*n*)	Change category^a^ % (*n*)
NRKW		26	58 (15)	42 (11)	Behav: 71%, Group: 17%, Speed: 12%
	DP	7	57 (4)	43 (3)	Behav: 100%
	PWSD	19	58 (11)	42 (8)	Behav: 60%, Group: 23%, Speed: 17%
DP		3	100 (3)	0 (0)	Not applicable
PWSD		5	40 (2)	60 (3)	Behav: 28%, Group: 28%, Speed: 44%

^a^ Change category refers to whether a change was in behavioral state (defined in Appendix A), group composition (grouped vs. dispersed), or group speed (fast, slow, or mixed).

### 3.4 Approach Direction and Swimming Position

Only porpoises and dolphins were observed approaching and initiating interactions; we never witnessed northern resident killer whales initiating any interactions. Since data were collected opportunistically, the approach direction was recorded for only 12 out of 39 interactions (*n*_porpoise_ = 4, *n*_dolphin_ = 8; see Appendix B). Observations implied that the approach direction of porpoises and dolphins initiating interactions with killer whales was highly variable (Appendix F).

**FIGURE 8:**
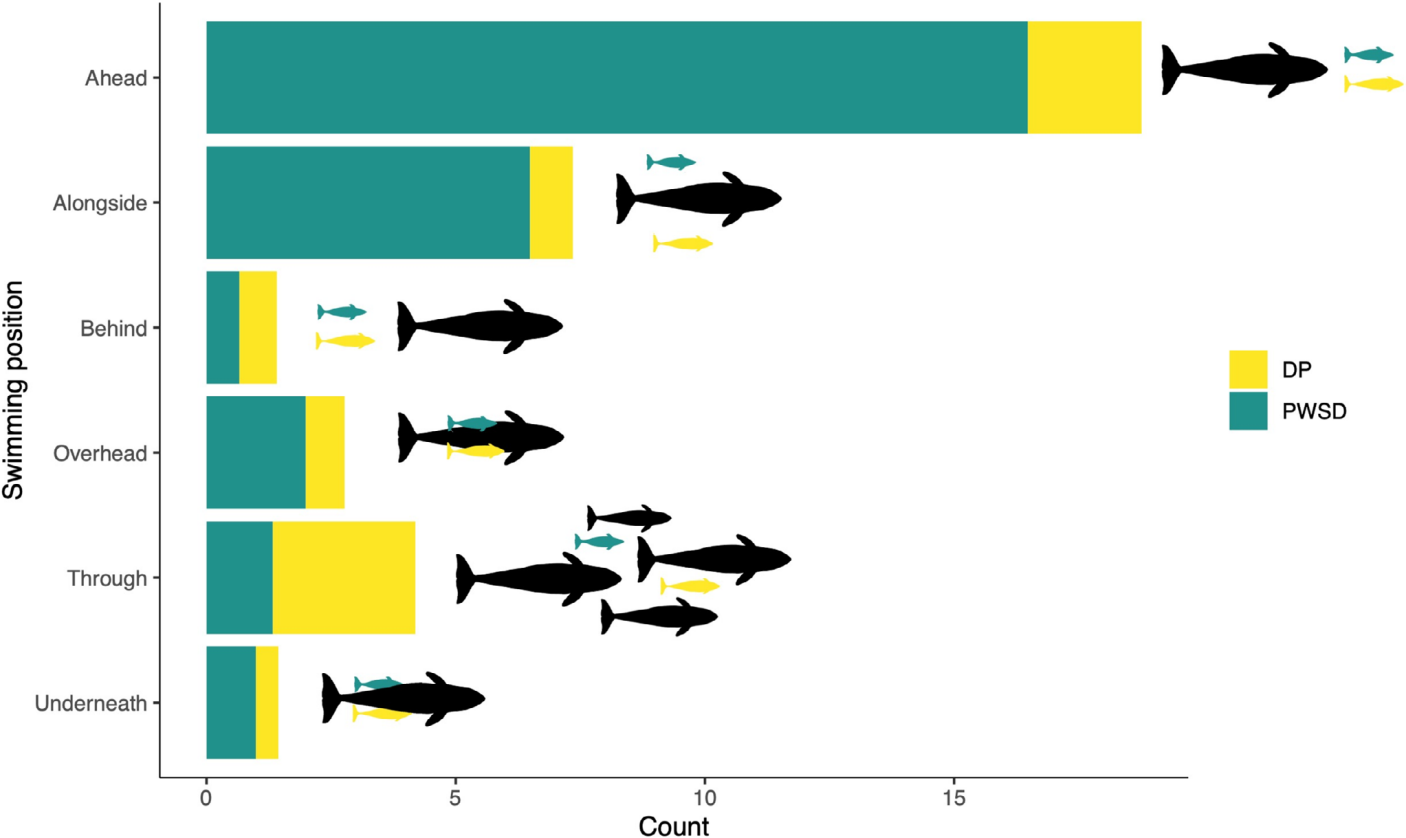
Swimming position of Dall's porpoises (DP, yellow) and Pacific white‐sided dolphins (PWSD, turquoise) relative to northern resident killer whales (black) during interspecific interactions recorded July to September of 2018 to 2021 off northeastern Vancouver Island, BC, Canada. Values indicate number of individual interactions where swimming position was recorded. Interactions where porpoises and/or dolphins were swimming in multiple positions throughout the interaction were equally divided, with the combined positions totalling to 1.

The swimming position of porpoises and dolphins relative to killer whales was noted in 36 out of 39 interactions (*n*_porpoise_ = 8, *n*_dolphin_ = 28; see Appendix B). Dolphins repeatedly swam ahead of or alongside killer whales during interactions, while porpoises swam either ahead of or through killer whale groups (Figure 8; see example in Video S4). On five occasions, dolphins and porpoises were observed swimming quickly through a group of killer whales before circling back to rejoin the slower‐moving group. During interactions, porpoises and dolphins regularly stayed at the surface when killer whales took long dives (five minutes or more), with two exceptions: one involved a killer whale holding and diving with a fish (see Video S5), and the other occurred when a pregnant adult female and her 6‐year‐old daughter dove at depth. Longer dives than the median duration of respiratory and foraging dives by northern residents (Wright et al. 2017) were noted during 10% (3 out of 31) of dolphin interactions (Appendix B).

## 4 Discussion

In this study, we documented the characteristics of opportunistically collected visual observations of Dall's porpoise and Pacific white‐sided dolphin interactions with northern resident killer whales. To our knowledge, our observations provide the most detailed accounts to date of interactions between Dall's porpoises and Pacific white‐sided dolphins with northern residents in the northeastern Pacific. Additionally, this is the first study to use drones to describe non‐predatory interspecific interactions involving resident killer whales and small cetaceans. Due to the opportunistic nature of the data and details recorded, it is difficult to draw definitive conclusions regarding the functional drivers behind the formation of the interactions observed. However, our observations provide support for the following species‐specific advantages hypothesized to drive the formation of mixed‐species groups: (1) antipredator advantages, (2) hydrodynamic advantages, (3) foraging advantages, and (4) social advantages.

### 4.1 Antipredator Advantages

The antipredator hypothesis suggests that mixed‐species groups form as a mechanism to reduce predation risk through increased safety in numbers (Lehtonen and Jaatinen 2016; van Langevelde et al. 2022), earlier predator detection (Seppänen et al. 2007; Huang et al. 2011; Magrath et al. 2015; van Langevelde et al. 2022), improved predator deterrence (Chiver et al. 2017; Pitman et al. 2017), and enhanced predator avoidance (FitzGibbon 1994; Godin and Davis 1995). Northern resident killer whales are apex predators and consequently are not expected to receive any antipredator advantages during interspecific interactions. Porpoises and dolphins could, on the other hand, be expected to benefit from the observed interactions with killer whales through reduced risk of predation by Bigg's killer whales.

Although large groups are easier for predators to detect, an individual's predation risk can be reduced as group size increases (i.e., the “dilution effect,” Foster and Treherne 1981; Lehtonen and Jaatinen 2016). Stensland et al. (2003) proposed that individuals may experience increased safety by staying with mixed‐species groups until more conspecifics are located. While it is unclear if other groups of porpoises and dolphins were within acoustic range of individuals involved in killer whale interactions, we did observe one account of a lone Pacific white‐sided dolphin interacting with a group of northern residents before joining other dolphins (see interaction 31/08/2019 in Appendix B).

Antipredator tactics are not only energetically costly for species attempting to escape predation, but individuals may also incur additional costs associated with specific antipredator defenses (Ives and Dobson 1987). We did not witness physical contact by northern resident killer whales, only mildly antagonistic behaviors (i.e., tail slapping and lunging; see Appendix B for details). The parallel population of resident killer whales in the south, however, is known to harass porpoises (Jefferson et al. 1991; Ford et al. 1998; Giles et al. 2023). Observations by Ford et al. (1998) and Giles et al. (2023) describe southern resident killer whales chasing, raking (causing temporary or permanent parallel scratches with teeth), pushing, carrying, and even killing (but never consuming) Dall's porpoises and harbor porpoises (*Phocoena phocoena vomerina*) in southern British Columbia as a form of play that helps individuals learn and fine‐tune hunting skills. Incidents of porpoise harassment by northern resident killer whales have been documented at least six times, one of which was suspected to have resulted in the death of the porpoise involved (Ford et al. 1998; Giles et al. 2023). Harassment of Pacific white‐sided dolphins by northern residents has not previously been reported in the literature; however, agitated behavior exhibited by the whales during interspecific interactions has been described (Giles et al. 2023). For porpoises, the smallest of the three species (Ford 2014), the potentially greater risk of interacting with northern resident killer whales may explain the shorter interaction durations recorded. In comparison to dolphin interactions, which were observed more frequently and lasted longer, the costs of interacting with northern resident killer whales may not be sufficient to deter dolphins.

One likely antipredator benefit that may justify the risks of approaching potentially harmful resident killer whales is the valuable knowledge porpoises and dolphins may gain from predator inspections. Dugatkin and Godin (1992) suggested that predator inspections can provide animals with information to avoid predation by examining how a predator hunts, moves, and acts, which is information they can use when and if attacked. Resident killer whales are morphologically analogous to Bigg's killer whales and are thus expected to have similar swimming speeds, agility, and strength. By associating with northern residents, one could presume that porpoises and dolphins are likely to gain some knowledge of the maneuverability of their main predator over multiple interactions. Thomson's gazelles (*Gazella thomsoni*) are known to spend a sizeable part of their day stalking and learning about their large feline predators (FitzGibbon 1994). As both porpoises and dolphins interacted with killer whales of varying ages and sexes during various behavioral states for up to 105 min, it is possible to infer that they had the opportunity to learn about their predator in a low‐risk environment by closely observing their relative.

The most compelling antipredator advantage Dall's porpoises and Pacific white‐sided dolphins may experience by associating with northern resident killer whales is the ensuing protection from Bigg's killer whales. Northern residents were never observed interacting with Bigg's, also present in Johnstone Strait during the study period (Appendices C and D), and such interactions are almost entirely missing from the literature. The only published report of a sustained close association that we are aware of was aggressive; when a group of residents briefly attacked a smaller group of Bigg's (Ford and Ellis 1999). Accounts of Bigg's avoiding groups of residents have been periodically reported in the northeastern Pacific (A. B. Morton 1990; Baird and Dill 1995). We observed comparable behaviors on several occasions during this study (Appendices D and E), where the two ecotypes passed within a kilometer of each other without intermixing, either maintaining non‐intersecting courses or altering course to avoid one another. One of us (L. Barrett‐Lennard) observed residents and Bigg's each forming tight groups, ceasing vocal activity, and moving away from each other when they came within several kilometers on three occasions in Prince William Sound, Alaska in 1990–91. Based on the clear reports of avoidance between the two ecotypes, associating with northern residents almost certainly reduces the risk of Dall's porpoises and Pacific white‐sided dolphins being preyed upon by Bigg's, whether or not such protection is realized or sought.

While there is a trade‐off between the benefits and costs of antipredator defenses, this balance can shift over time as the efficiency of the behavior increases (Ives and Dobson 1987). For porpoises and dolphins, that cost can be reduced by effectively recognizing groups of resident and Bigg's killer whales. Because northern resident and Bigg's killer whales are sympatric and common in our study area, it is likely that all mature dolphins and porpoises in British Columbia have been repeatedly exposed to both ecotypes, and that individuals engaged in interactions with residents are able to distinguish between the two. This is supported by our observations of both species approaching northern residents from directly ahead. Vocalizations have been proposed as a safe signal that dolphins and porpoises use to recognize the two ecotypes, as resident killer whales are highly vocal in comparison to Bigg's and have acoustically distinct calls (Jefferson et al. 1991; Ford et al. 1998) and echolocation pulses (Barrett‐Lennard et al. 1996). While we did not record the soundscape during encounters, collecting acoustic recordings before and during interspecific interactions, in addition to comprehensive behavioral narratives, will elucidate how and when porpoises and dolphins choose to interact with northern residents.

### 4.2 Hydrodynamic Advantages

One aspect of living in a three‐dimensional marine environment is that an individual's hydrodynamic efficiency whilst swimming can be influenced by its location relative to others in the group (Bumann et al. 1997; Weihs 2004). Both porpoises and dolphins were frequently sighted swimming ahead of northern resident killer whales, and occasionally swam in other positions (i.e., alongside, behind, overhead, and underneath). Swimming position can serve as an antipredator tactic for group‐swimming individuals. For example, Bumann et al. (1997) found that lake chub ( *Semotilus atromaculatus* ) were safest from ambush predators when swimming in the rear and middle of the shoal. Consequently, the predominantly frontward swimming position observed does not reinforce antipredator benefits for porpoises and dolphins during interactions but may suggest an alternative benefit. An experimental study by Ristroph and Zhang (2008) revealed that the leading individuals in schooling fish experienced reduced drag compared to those downstream, which may help explain the observed swimming positions of porpoises and dolphins. During interactions, porpoises and dolphins may also attempt to draft beside or behind the killer whales, a technique employed by calves to surf alongside their mother's flank or underneath the tailstock to save energy and keep pace (Weihs 2004; Noren et al. 2008).

While drafting may provide hydrodynamic benefits to porpoises and dolphins that interact with northern resident killer whales, it also comes with an increased risk of being accidentally or purposely struck by a killer whale's tail fluke. Although we did not observe porpoises or dolphins being struck by northern resident killer whales, we did witness an adult male lunge at a group of dolphins swimming directly ahead (see Video S3 and interaction on 06/09/18 in Appendix B). In most encounters, porpoises swam through killer whale groups, while dolphins spent a considerable amount of time swimming ahead or alongside groups. A significantly different characteristic between dolphin and porpoise interactions with northern resident killer whale was that porpoises only interacted with small groups of killer whales compared to dolphins. Interacting with smaller killer whale groups and swimming in different positions may offset the cost of potentially being struck for porpoises. However, Dall's porpoises are known to commonly bowride in the pressure wave of vessels for short periods (Jefferson 1991; Hanson and Baird 1998), which supports the shorter interaction durations recorded. This suggests that while porpoises may gain hydrodynamic advantages during killer whale interactions, these benefits may not be significant enough to justify prolonged interactions or engaging with larger groups that increase the likelihood of harm.

### 4.3 Foraging Advantages

The foraging advantage hypothesis implies that the formation of interspecific interactions increases the foraging success of one or both of the interactors (Jourdain and Vongraven 2017; Syme et al. 2021). This increase may result from shared knowledge about the location of food resources (Norconk 1990), increased search efficiency (Pitcher et al. 1982), and/or enhanced prey detection and capture through cooperative foraging (Stensland et al. 2003). However, our observations provided no concrete evidence that porpoises or dolphins experience increased foraging opportunities during killer whale interactions as no salmon, the primary prey of northern residents when in Johnstone Strait (Ford et al. 2000), were visibly consumed or caught. That said, resident killer whales routinely transport prey caught at depth to the surface to consume, either independently or through social sharing (Ford and Ellis 2006; Wright et al. 2016), and it is possible that porpoises and dolphins collect or steal fish or scraps during these events. The frontward swimming preference observed also likely offers better foraging opportunities. It is doubtful that northern resident killer whales, which specialize on Chinook salmon (Ford et al. 2010), benefit from foraging opportunities during interactions with porpoises and dolphins, which are attracted to species of schooling fish (Heise 1997; A. Morton 2000; Nichol et al. 2013). Furthermore, it is at least plausible that porpoises and dolphins swimming directly ahead of the whales may reduce the killer whales' ability to locate or successfully pursue salmon. This theory of reduced foraging efficiency might explain the one occurrence of antagonistic lunging observed by a foraging adult male northern resident (see Video S3 and interaction on 06/09/18 in Appendix B).

### 4.4 Social Advantages

Under the social advantage hypothesis, reproductive practice, social status, bond formation, physical and social development, and alloparental care are potential social benefits of mixed‐species groups (Herzing and Johnson 1997; Stensland et al. 2003; Hill et al. 2017; Mrusczok et al. 2023). A study by Syme et al. (2023) found that social advantages were the best‐supported explanation for mixed‐species groups of Australian humpback ( *Sousa sahulensis* ) and Indo‐Pacific bottlenose dolphins ( *Tursiops aduncus* ) off Western Australia, with individuals benefiting from social‐sexual skill development and alloparental care. We did not make observations of, nor receive reports of behaviors consistent with interspecific alloparental care or sexual behavior in this study. On the contrary, the incident of tail slapping by the slow traveling, mother‐calf pair (see Figure 7 and interaction on 08/07/2021 in Appendix B) supports the opposite. The female may have instead been protecting her calf from disturbance as tail slapping is thought to have several functions, one of which is to communicate annoyance or aggression (Ford et al. 2000; Stensland and Berggren 2007). Alternatively, alloparental care may not have been observed due to the substantial size difference between resident killer whales (4.7 to 7.25 m) and both Dall's porpoises (1.52 to 2.35 m) and Pacific white‐sided dolphins (2.4 to 2.5 m; Ford 2014), a difference not observed between Australian humpback and Indo‐Pacific bottlenose dolphins (Baker 1990).

Resident killer whales were not always antagonistic in behavior; they often appeared neutral (no obvious reaction), and several occurrences of affiliative behavior (positive social reaction that was not aggressive or predatory) were also documented. While most of the affiliative observations involved young killer whales that are likely to engage in “play” activity that has no specific reward or consequence (Hill et al. 2017), interacting may also help them develop valuable physical, cognitive, and emotional skills (Struhsaker 1981; Vanderschuren and Trezza 2014). The interactions exhibiting “play” behavior were some of the longest interspecific interactions recorded for both porpoises and dolphins. Notably, all porpoise interactions greater than 10 min were associated with the presence of killer whale calves. Similar instances of Australian humpback and Indo‐Pacific bottlenose dolphin calves engaged in apparent play behavior were recorded by Syme et al. (2023).

Interactions with porpoises and dolphins often resulted in changes to northern resident killer whale behavior, including a reduction in speed, increased group dispersion, or a shift in social activity from resting to traveling. It is unclear whether these are typical behavioral changes exhibited by northern resident killer whales as they move through the study area—such as traveling to different sites to forage or commencing travel after a period of rest—or are the result of interactions with porpoises and dolphins. If these changes are due to interactions, our observations alone are insufficient to support the idea that northern resident killer whales gain social benefits from interacting with porpoises and dolphins in the northeast Pacific. In fact, northern resident killer whales may derive no benefits from these interactions. However, hindering such interactions may impose a greater cost on the killer whales than any negative implications of the interactions themselves.

## 5 Conclusion

This study provides the most detailed description of Dall's porpoise and Pacific white‐sided dolphin interactions with northern resident killer whales to date and demonstrates that interactions between northern residents and these two small cetacean species are common in the coastal waters of western Canada. Dall's porpoises and Pacific white‐sided dolphins frequently approached and swam with northern resident killer whales. Mildly antagonistic behaviors by northern residents did not appear to deter dolphins or porpoises from continuing to travel and associate with the whales, nor did they appear to affect the duration of interactions.

Despite not being able to conclude the exact drivers of the interspecific interactions observed, it appears that Dall's porpoises and Pacific white‐sided dolphins experience benefits from interacting with resident killer whales that outweigh the small risk of being harmed by them; mainly by reducing the risk of being approached by Bigg's killer whales. Bigg's killer whales appeared to actively avoid northern residents in the study area, thus one key benefit that Pacific white‐sided dolphins and Dall's porpoises experience by swimming with residents is a period of protection from their principal predator. We suggest that porpoises and dolphins may also have the opportunity to learn about the speed, agility, acceleration ability, and other behaviors of resident killer whales that may benefit them if they are pursued or attacked by Bigg's, which are morphologically similar. Additional benefits suggested by our findings include reduced costs of locomotion resulting from drafting (“hydrodynamic hitch‐hiking”), the opportunity to obtain scraps from prey‐sharing killer whales, and/or social advantages not yet understood. The differences in interaction characteristics between Dall's porpoises and Pacific white‐sided dolphins imply species‐specific benefits, with social advantages being the more likely trigger for Dall's porpoise‐northern resident interactions. No clear advantages of interspecific interactions were found for northern resident killer whales, which never initiated an interaction in the present study.

The work presented here contributes to a growing body of research highlighting the potential of drone footage in behavioral studies of cetaceans (e.g., Torres et al. 2018; Weiss et al. 2021; Ramos et al. 2023; Hamel et al. 2025) and provides new insights into killer whale interspecific interactions not previously described. Future systematic studies that investigate the hypotheses discussed are required to better understand the drivers and functions of interspecific interactions between northern resident killer whales and small cetaceans in the North Pacific.

## Author Contributions

**Brittany C. Visona‐Kelly:** conceptualization (equal), data curation (lead), formal analysis (lead), funding acquisition (equal), investigation (equal), methodology (lead), project administration (lead), visualization (lead), writing – original draft (lead), writing – review and editing (lead). **Lance G. Barrett‐Lennard:** conceptualization (equal), funding acquisition (equal), investigation (equal), methodology (supporting), project administration (supporting), supervision (lead), writing – original draft (supporting), writing – review and editing (supporting).

## Conflicts of Interest

The authors declare no conflicts of interest.

## Supporting information


Appendix S1.
**Video S1.** Two Dall’s porpoise approaching and circling a logging (i.e., resting) northern resident killer whale, 40‐year‐old adult female A43, on July 8, 2021, in Johnstone Strait, BC, Canada.
**Video S2.** Dall’s porpoises traveling and socializing with northern resident killer whale A69 (24‐year‐old adult female) and her newborn calf A126 on July 8, 2021, in Johnstone Strait, BC, Canada.
**Video S3.** Aggressive lunge displayed by 24‐year‐old adult male northern resident killer whale A61 during a Pacific white‐sided dolphin interaction in Blackfish Sound, BC, Canada on September 6, 2018.
**Video S4.** Example of Pacific white‐sided dolphins and Dall’s porpoises swimming with northern resident killer whales in the northeastern Pacific.
**Video S5.** Pacific white‐sided dolphins approaching a northern resident killer whale carrying a fish in Blackfish Sound, BC, Canada on August 13, 2020.

## Data Availability

The data supporting the findings of this study are available in Appendix B and Appendix E. Selected captured photos are available within this article and example videos at https://doi.org/10.5061/dryad.2bvq83c2c. Encounter details for example videos can be found in Appendix S1. Other photos and videos are available upon request.

## Appendix A Definitions of Behavioral State of Whales, Dolphins, and Porpoises Based on Ford (1989), Baird and Dill (1995), and Barrett‐Lennard et al. (1996).

**Table:**

Behavioral state	Description
Slow travel (ST)^a^	
Grouped (−G)	All individuals in the group moving directionally or non‐directionally at constant speeds of 5 knots or less
Dispersed (–D)	Group is spread out, moving directionally or non‐directionally at speeds of 5 knots or less. Matriline split into smaller groups
Fast travel (FT)^a^	
Grouped (−G)	All individuals in the group moving directionally or non‐directionally at speeds greater than 5 knots
Dispersed (–D)	Group is spread out, moving directionally or non‐directionally at speeds greater than 5 knots. Matriline split into smaller groups
Milling (M)	Individuals making non‐directional movements, taking longer duration dives. Surfacing not coordinated with other individuals. Includes individuals actively feeding (i.e., prey tissue, scales, blubber, etc. visible or prey sharing evident between individuals)
Socializing (S)	Interacting with other individuals through touching, rolling, rubbing, and/or playful behavior underwater. Can be sexual or in combination with surface active behaviors (i.e., individuals display breaching, spy‐hopping, cartwheeling, dorsal slapping, pectoral slapping, and/or tail slapping behavior on the water's surface)
Resting (R)	Individual, or horizontal line of multiple individuals, remaining at surface for period of time before a shallow dive. No movement while at the surface. Includes resting line of individuals surfacing in unison in a directional movement at a slow constant pace

^a^ Animal speed was considered as the relative speed the vessel had to maintain to keep pace with traveling individual(s).

## Appendix B Details of Interactions Between Northern Resident Killer Whales (NRKW) and Pacific White‐Sided Dolphins (PWSD) and/or Dall's Porpoise (DP) Off Northeastern Vancouver Island, BC, Canada From 2018 to 2021. See Appendix A for Behavioral State Descriptions.

**Table:**

Date (date/month/year)	Location (lat., long.)	Behavioral State of NRKW		Behavioral state of other species		Interaction details	Duration (min)
		Before	During	Before	During		
08/08/2018	50.556, −126.796	NR	ST‐D	NR	ST‐G	8 PWSD slowly swam ahead, alongside, and behind 3 NRKW—A75 (16‐year‐old female), A101 (5‐year‐old female), and A113 (1‐year‐old female)	5
08/08/2018	50.538, −126.685	ST‐G, M	NR	FT‐G	ST‐G	3 PWSD slowly swam ahead of 7 NRKW (A50s, A54s). Salmon were present in the area	4–5
11/08/2018	50.757, −127.064	NR	NR	NR	ST, FT	5 PWSD slowly, and occasionally quickly, swam with NRKW (A34s, A42s, I4s)	NR
12/08/2018	50.532, −126.667	ST‐G, M	ST‐D, M	NR	PWSD: ST‐G, S. DP: FT‐G, S	8–10 PWSD slowly swam ahead (mainly), alongside, and behind NRKW (A50s, A54s). 4–5 DP swam quickly through and ahead of NRKW, occasionally circling back behind group before traveling on. PWSD and DP socialized with one another	5–8
22/08/2018	50.720, −127.100	NR	NR	NR	FT	20 PWSD swam ahead and alongside 24 NRKW (A25s, A50s, A54s, I4s, I16s). NRKW spread over several miles	NR
24/08/2018	50.534, −126.742	ST‐D, M	FT‐D	NR	FT	8 NRKW (A25s, A34s) spread over several miles. PWSD with small groups. PWSD swam ahead and alongside NRKW. NRKW took long dives	NR
29/08/2018	50.628, −126.814	FT‐G	ST‐D, M	NR	ST‐G, S	5 PWSD slowly swam ahead and above 18 NRKW (A50s, A54s)—A84 (13‐year‐old male) in particular. PWSD did not follow NRKW on deep dives. Longer dives recorded when PWSD present	5–10
01/09/2018	50.665, −126.760	ST‐D, M	ST‐D, M, S	NR	ST‐G, S	3 PWSD swam in circles playing with NRKW A118 (new 2018 female calf) for 3–5 min before A118 joined A86 (12‐year‐old pregnant female) and A108 (4‐year‐old male). PWSD traveled ahead of the group	8–12
06/09/2018	50.728, −126.936	ST‐D, M, S	ST‐D, M, S	ST‐G, FT‐G, S	ST‐D	20 NRKW (A50s, A54s) spread out milling. 20–30 PWSD in the area within 200 m of the tightly grouped NRKW. PWSD approached and traveled with smaller groups of NRKW for short durations	90–120
06/09/2018	50.614, −126.767	ST‐D, M	ST‐D, M	NR	FT‐G, S	7–9 PWSD swam ahead and above 1 NRKW, A61 (24‐year‐old adult male). A61 lunged towards the PWSD swimming directly in front. A61 took long, deep dives during interaction. PWSD did not follow during dives	20–25
03/08/2019	50.555, −126.801	NR	ST‐D	ST‐G, FT‐G	ST‐G, FT‐G	2–3 DP swam ahead and behind 5 NRKW (A23s)	NR
03/08/2019	50.554, −126.732	ST‐D, M	ST‐D, M	FT‐G	FT‐G	3 DP approached 1 NRKW, A69 (22‐year‐old adult female), and swam ahead for the duration of the interaction. DP would swim faster than A69 and circle back to swim in front	5
07/08/2019	50.545, −126.689	NR	ST‐D, M	ST‐G, M	FT‐D, S	8 PWSD swam ahead, above, and alongside 1 NRKW, I107 (14‐year‐old male). I107 took regular dives. PWSD did not dive with I107, staying near the surface until the NRKW resurfaced then continued traveling in front	12
10/08/2019	50.704, −126.931	ST‐D, S	ST‐D, S	NR	FT‐G	2 PWSD approached 2 NRKW, A101 (6‐year‐old female) and A113 (2‐year‐old female) once they started displaying surface‐active behaviors. PWSD swam through and ahead of the group	3–5
10/08/2019	50.736, −127.009	NR	ST‐D	NR	ST‐G	9 PWSD swam ahead of 3 NRKW—A106 (5‐year‐old male) and A86 (12‐year‐old female) with new 2019 calf A120 in tow	5
11/08/2019	50.766, −127.110	ST‐G	ST‐G	NR	FT‐G	5 PWSD approached 8 NRKW (A50s, A54s) and swam ahead of the group for several minutes before moving on through the area	3–5
22/08/2019	50.709, −127.139	ST‐G	ST‐G	NR	FT‐G	4–5 PWSD approached 7 NRKW (A42s) from behind. PWSD swam ahead and alongside NRKW until they dove. PWSD continued swimming in the same direction until the NRKW surfaced again, then circled back and swam in front of the group	5–7
22/08/2019	50.672, −126.672	ST‐G	ST‐G	FT‐G	FT‐G	2–3 PWSD approached 7 NRKW (A42s) from the side. PWSD swam ahead and alongside NRKW; often swimming far ahead then circling back and quickly swimming in front of group. PWSD stayed with NRKW until they dove	3–5
31/08/2019	50.545, −126.689	M, R	ST‐D, M	NR	FT‐D, S	6–8 DP swam through, above, and alongside 2 NRKW (I4s, I27s, I65s, I16s), circling around at fast speeds with no apparent direction of travel. DP displayed social/playful behavior	5–8
31/08/2019	50.550, −126.698	R, S	ST‐D	NR	FT‐D	3 NRKW (A54s) rested in a group for several minutes before dispersing and commencing in social/playful and surface‐active behaviors. 5 DP approached NRKW after they were surface active, swam through group then moved on	1–3
31/08/2019	50.554, −126.728	ST‐G	ST‐D	NR	ST‐D	1 PWSD approached an all‐male group of 4 NRKW (I77, I98, I106, I129) from the front, swam through the group, then traveled directly ahead of I98 (17‐year‐old‐male) for several minutes before continuing and joining 5 PWSD	5
08/08/2020	50.704, −127.012	NR	FT‐D, S	NR	FT	3–5 PWSD swam quickly ahead of 8 NRKW (R13s, R18s), circling back through the group to stay with them. Mainly in front of R37 (23‐year‐old adult male). R57 (9‐year‐old whale) became surface active when the PWSD were swimming closely alongside	5
13/08/2020	50.605, −126.852	ST‐D, M	ST‐D, M	NR	FT	2–5 PWSD swam ahead and alongside 12 NRKW (A50s, A54s). One PWSD stayed around a whale holding a fish; when the whale dove the PWSD followed	5
15/08/2020	50.527, −126.637	ST‐G	ST‐G	NR	FT	1–2 PWSD approached large group of 15 NRKW (I4s, I13s, I16s, I65s) traveling in a line. PWSD swam ahead and alongside group; in the middle of the line	2–5
15/08/2020	50.513, −126.597	ST‐G, R	ST‐G, R	NR	ST	1–2 PWSD approached resting group of 15 NRKW (I4s, I13s, I16s, I65s) from behind. Slowly traveled with the resting line, swimming under, behind, and ahead of the group	2–5
15/08/2020	50.517, −126.607	R, S	ST‐G, S	NR	ST	15 NRKW (I4s, I13s, I16s, I65s) dispersed; some logging, others socializing—rolling on one another and spyhopping. 1–2 PWSD swam with NRKW once they started traveling	2–5
16/08/2020	50.677, −126.884	ST‐D, M	ST‐D, M	NR	FT‐G	3 NRKW (I65s) slowly traveled and foraged before interaction. One whale had a fish in its mouth. 2 DP approached I65 (30‐year‐old adult female) from behind, traveled quickly through the group and moved on	2–5
28/08/2020	50.583, −126.807	NR	NR	ST	ST	200–300 PWSD swam by group of 25 NRKW (R5, R30, C6s, C13s, D9, D11s, D12s, D19). PWSD swam alongside the group several times, no more than a couple of minutes, before leaving the whales	2–5
30/08/2020	50.576, −126.912	ST‐D, M	ST‐D, S	NR	FT‐D, S	5–6 PWSD swam under, above, ahead of 3 KWs—I51 (34‐year‐old adult female), new 2020 calf I164, and I144 (6‐year‐old whale). Several PWSD swam closely and socialized with I144 and I164. I164 was playful, swimming away from its mother I51 and sibling I144 to swim with the PWSD. PWSD mainly swam in front of the 7 NRKW (I16s). Majority of PWSD stayed with I164, even when it was swimming with I51	15–20
31/08/2020	50.557, −126.834	NR	ST‐G	NR	NR	PWSD swam closely ahead and alongside 12 NRKW (C6s, C10s)	NR
10/09/2020	50.549, −126.830	ST‐D, M	ST‐D, M	NR	FT	3–5 PWSD quickly traveled with pregnant A69 (23‐year‐old adult female) and A109 (6‐year‐old female). PWSD swam under, above, and ahead of 5 NRKW (A23s). PWSD dove at depth with NRKW	5–10
10/09/2020	50.549, −126.830	NR	ST‐D, M	NR	PWSD: FT. DP: FT	3–5 PWSD and 2–3 DP quickly swam through, alongside, and ahead of spread‐out group of 8 NRKW (A23s, A25s). PWSD and DP interacted with the NRKW on and off for duration of the encounter	20–40
07/07/2021	50.583, −126.700	ST	ST	NR	FT	PWSD and 5–6 DP approached and swam through, alongside, and ahead of 17 NRKW (A23s, A25s, A54s)	NR
08/07/2021	50.544, −126.776	ST‐D	ST‐D, S	NR	FT‐G, S	15–20 DP swam ahead, behind, above, and under A69 (24‐year‐old adult female) and newborn calf A126. DP interested in A126, swimming closely in a playful manner. A126 associated with DP, swimming away from A69 at times to follow quick moving DP. A69 tail slapped several times when numerous DP were swimming around her and A126	25
08/07/2021	50.544, −126.776	R	R	NR	S	Two DP approached logging A43 (40‐year‐old adult female) – approached from the front and circled around before returning to other DP swimming with A69 and A126	5
18/08/2021	50.626, −126.763	M	M	NR	FT	8–10 PWSD swam with 3 foraging NRKW (I27s), swimming quickly ahead of I107 (17‐year‐old male) and I77 (24‐year‐old adult male)	10
22/08/2021	50.619, −126.822	ST‐G	ST‐G	NR	ST	10–20 PWSD swam ahead and alongside 4 NRKW – I65 (31‐year‐old adult female), I122 (14‐year‐old male), I145 (6‐year‐old juvenile), and I165 (one‐year‐old calf)	15
23/08/2021	50.549, −126.809	NR	ST‐D, FT‐D, S	NR	FT‐G	4–5 DP swam ahead, above, under, alongside, and through group of 3 NRKW (A42s). KW began swimming quickly with the DP	10
23/08/2021	50.538, −126.847	NR	ST‐D, M	NR	FT‐G	3–5 PWSD swam quickly with 2 NRKW – A85 (16‐year‐old female) and A121 (two‐year‐old calf). PWSD mainly ahead and alongside A121	10
23/08/2021	50.551, −126.834	NR	R	NR	FT	8–10 PWSD swam ahead of resting group of 10 NRKW (A42s, I4s)	5
25/08/2021	50.479, −126.143	NR	ST‐G	NR	FT	8–10 PWSD swam ahead of 7 NRKW (A42s)	5
27/08/2021	50.521, −126.699	ST‐G	FT‐D, ST	NR	ST	Large grouped of 23 NRKW were slowing traveling before 20–30 PWSD were present. Group split when PWSD appeared—A35s and A73s traveled quickly east. PWSD swam ahead of 9 slowly traveling KWs (A23s, A25s)	5

Abbreviation: NR, not recorded.

## Appendix E Sightings of Northern Resident Killer Whales (NRKW) and Bigg's Killer Whales (BKW) Recorded on the Same Day Off Northeastern Vancouver Island, BC, Canada During Annual Fieldwork From 2018 to 2021. See Appendix A for Behavioral State Descriptions.

**Table:**

Date (date/month/year)	Time (h/min)	Location (lat., long.)	Ecotype	Group ID	Group size (minimum)	Behavioral state
08/08/2018	11:03	50.6096, −126.787	BKW	T046s	5	FT
08/08/2018	16:12	50.556, −126.797	NRKW	A42s	7	ST
15/08/2018	08:51	50.481, −126.433	NRKW	A23s, A25s, A34s, A50s, A54s, I4s	25	ST
15/08/2018	12:31	50.610, −126.805	BKW	T087, T124s	5	ST
08/08/2019	10:05	50.574, −126.851	BKW	T077C, T077D	2	ST
08/08/2019	12:23	50.660, −126.855	NRKW	A50s, A54s, I4s, I27s, I35s	5	ST
10/08/2019	08:00	50.704, −126.931	NRKW	A50s, A54s, A25s, I4s	18	ST
10/08/2019	10:10	50.611, −126.828	BKW	T055s	4	ST
19/08/2019	09:02	50.522, −126.557	NRKW	A50s, A54s, I4s	15	NR
19/08/2019	11:25	50.698, −126.860	BKW	T018s	4	NR
19/08/2019	18:07	50.840, −127.536	BKW	T060D, T60E	2	NR
24/08/2019	10:59	50.681, −126.939	NRKW	A50s, A54s, A42s	15	ST
24/08/2019	11:50	50.597, −126.755	BKW	T002B, T059, T060s	7	NR
03/09/2019	11:37	50.836, −127.752	NRKW	NR	10	ST, M
03/09/2019	15:16	50.595, −127.024	BKW	T010s	2	ST
12/08/2020	07:52	50.559, −126.686	NRKW	A50s, A54s	8	ST, S
12/08/2020	09:29	50.511, −126.546	BKW	T002Cs	4	FT
25/08/2020	10:32	50.512, −126.670	NRKW	A50s, A54s	10	ST
25/08/2020	12:40	50.730, −126.720	BKW	T055B, T055C	2	M
30/08/2020	09:32	50.731, −126.974	NRKW	I16s	5	ST
30/08/2020	12:50	50.573, −126.896	BKW	NR	3	ST
07/07/2021	10:07	50.541, −126.763	BKW	T036A2, T036A3, T137s	6	ST
07/07/2021	16:13	50.606, −126.750	NRKW	A23s, A25s, A54s	15	ST
07/08/2021	08:03	50.642, −126.792	NRKW	I4s, I27s, I65s	10	ST, S
07/08/2021	14:20	50.608, −126.988	BKW	T002Cs	4	ST
10/08/2021	08:15	50.613, −126.780	NRKW	I4s, I27s, I65s	10	ST, M
10/08/2021	13:30	50.584, −126.833	BKW	T060D, T060E	2	ST
15/08/2021	09:00	50.526, −126.705	NRKW	I4s, I27s, I65s	10	ST, FT
15/08/2021	11:46	50.612, −127.094	BKW	T041s, T109Cs, T121As	9	FT
16/08/2021	10:04	50.557, −126.762	NRKW	I27s	3	NR
16/08/2021	10:24	50.601, −126.742	BKW	T099s	4	ST
18/08/2021	08:53	50.556, −126.690	NRKW	I4s, I27s, I65s	10	ST
18/08/2021	15:59	50.613, −126.823	BKW	T046s	8	M
23/08/2021	09:20	50.672, −127.262	BKW	T065Bs	3	NR
23/08/2021	16:59	50.543, −126.776	NRKW	A25s, A42s, I4s, I27s, I65s	20	ST

Abbreviation: NR, not recorded.
